# Tuberculosis Diagnostic Delays in Indonesian Primary Care Pathways

**DOI:** 10.5334/aogh.5369

**Published:** 2026-07-16

**Authors:** Suherman Jaksa, Muhammad Fachri, Risky Akaputra, Nurmalia Lusida, Irna Hasanah, Raghib Filhaq

**Affiliations:** 1 Faculty of Public Health, Universitas Muhammadiyah Jakarta, South Tangerang, Indonesia; 2 Faculty of Medicine and Health, Universitas Muhammadiyah Jakarta, South Tangerang, Indonesia; 3 Faculty of Islamic Studies, Universitas Islam Jakarta, Jakarta, Indonesia

**Keywords:** tuberculosis, diagnostic delay, health equity, primary care, Indonesia, care cascade

## Abstract

*Background:* Timely tuberculosis diagnosis is essential for reducing transmission, preventable morbidity, and inequitable losses across the care cascade. In Indonesia, primary health centres are central to screening and diagnostic coordination, but delays may still occur after individuals with presumptive tuberculosis enter formal care.

*Objective:* To quantify post-registration diagnostic delay among people with presumptive tuberculosis in Indonesian primary care and identify demographic, clinical, and referral-pathway factors associated with delayed results, tuberculosis diagnosis, and treatment initiation.

*Methods:* We conducted a facility-based, register-based observational study using national presumptive tuberculosis register data from a primary health centre in Tasikmalaya City, West Java, Indonesia, covering 1 January to 31 December 2025. Diagnostic delay was defined as the interval from presumptive tuberculosis registration to the first recorded diagnostic result. Negative binomial regression estimated adjusted incidence rate ratios for delay, and logistic regression estimated adjusted odds ratios for prolonged delay, final diagnosis, and treatment initiation.

*Findings:* Among 503 presumptive tuberculosis episodes, 486 had complete delay data. Median diagnostic delay was 4 days (interquartile range, 1–11 days). Delays longer than 14 and 30 days occurred in 94 episodes (19.3%) and 37 episodes (7.6%), respectively. Longer delay was associated with age 65 years or older (adjusted incidence rate ratio, 1.46; 95% CI, 1.12–1.90), extrapulmonary disease (1.58; 95% CI, 1.21–2.07), and referral from another health facility (1.35; 95% CI, 1.01–1.82). Overall, 176 presumptive episodes (35.0%) resulted in a tuberculosis diagnosis; 158 diagnosed patients (89.8%) had documented treatment initiation.

*Conclusions:* Although most diagnostic results were recorded rapidly after registration, delays were concentrated among older adults, people with extrapulmonary disease, and referred patients. Strengthening register-based tracking, referral feedback, and escalation pathways for extrapulmonary tuberculosis may improve equitable continuity across primary care tuberculosis services.

## Introduction

Tuberculosis remains a major global health challenge and a leading infectious cause of preventable morbidity and mortality. Indonesia is among the countries with the largest tuberculosis burdens globally, with national estimates continuing to show a substantial gap between estimated incidence, diagnosis, notification, and complete treatment coverage [1, 2]. Because tuberculosis control depends on early detection, prompt initiation of treatment, and continuous follow-up, delays at any point in the diagnostic pathway can weaken both individual patient outcomes and population-level disease control [3–6]. Breakdowns between diagnosis and treatment initiation, often referred to as initial loss to follow-up, represent an important care cascade gap because patients may remain untreated despite receiving a diagnosis.

Primary health centres occupy a strategic position in Indonesia’s tuberculosis response. They screen people with symptoms or epidemiologic risk, register individuals with presumptive tuberculosis, coordinate specimen collection and laboratory testing, receive diagnostic results, initiate treatment, and support community follow-up according to national programme algorithms [4]. These facilities are therefore a critical interface between community case finding, public-private referral networks, and treatment initiation, particularly in settings where people may first seek care through multiple public, private, or informal providers [7, 8]. Current Indonesian TB guidelines emphasise rapid completion of diagnostic evaluation, particularly when molecular testing is available; however, no nationally standardised benchmark for post-registration diagnostic turnaround time is routinely reported.

Diagnostic delay is also an equity issue. Prior studies have shown that age, sex, disease site, referral pathway, migration, comorbidity, stigma, and provider type may influence whether symptoms are recognised, diagnostic tests are requested, results are returned, and treatment begins without interruption [9–16]. Most available studies, however, measure delay from symptom onset or first care seeking among confirmed tuberculosis patients. Less is known about the narrower but operationally important interval after a person has already been registered as having presumptive tuberculosis in primary care.

Presumptive tuberculosis refers to individuals presenting with symptoms, signs, or epidemiological risk factors suggestive of tuberculosis who require further diagnostic evaluation according to national and WHO guidance. Analysing these registers can identify groups who remain vulnerable to avoidable delay even after reaching primary care services. This study used routine presumptive tuberculosis register data from a primary health centre in West Java, Indonesia, to quantify post-registration diagnostic delay and examine demographic, clinical, and referral-pathway factors associated with delayed results, tuberculosis diagnosis, and treatment initiation. The outcome examined in this study reflects post-registration diagnostic delay and facility-level diagnostic turnaround time rather than total patient diagnostic delay.

## Methods

### Study design and setting

This facility-based, register-based observational study used data from the national presumptive tuberculosis register at a primary health centre in Tasikmalaya City, West Java, Indonesia. The dataset covered 1 January to 31 December 2025 and reflected routine fields used for presumptive tuberculosis registration, diagnostic testing, and care cascade monitoring. The manuscript was prepared in accordance with the Strengthening the Reporting of Observational Studies in Epidemiology reporting principles.

### Study population

**Figure 1:**
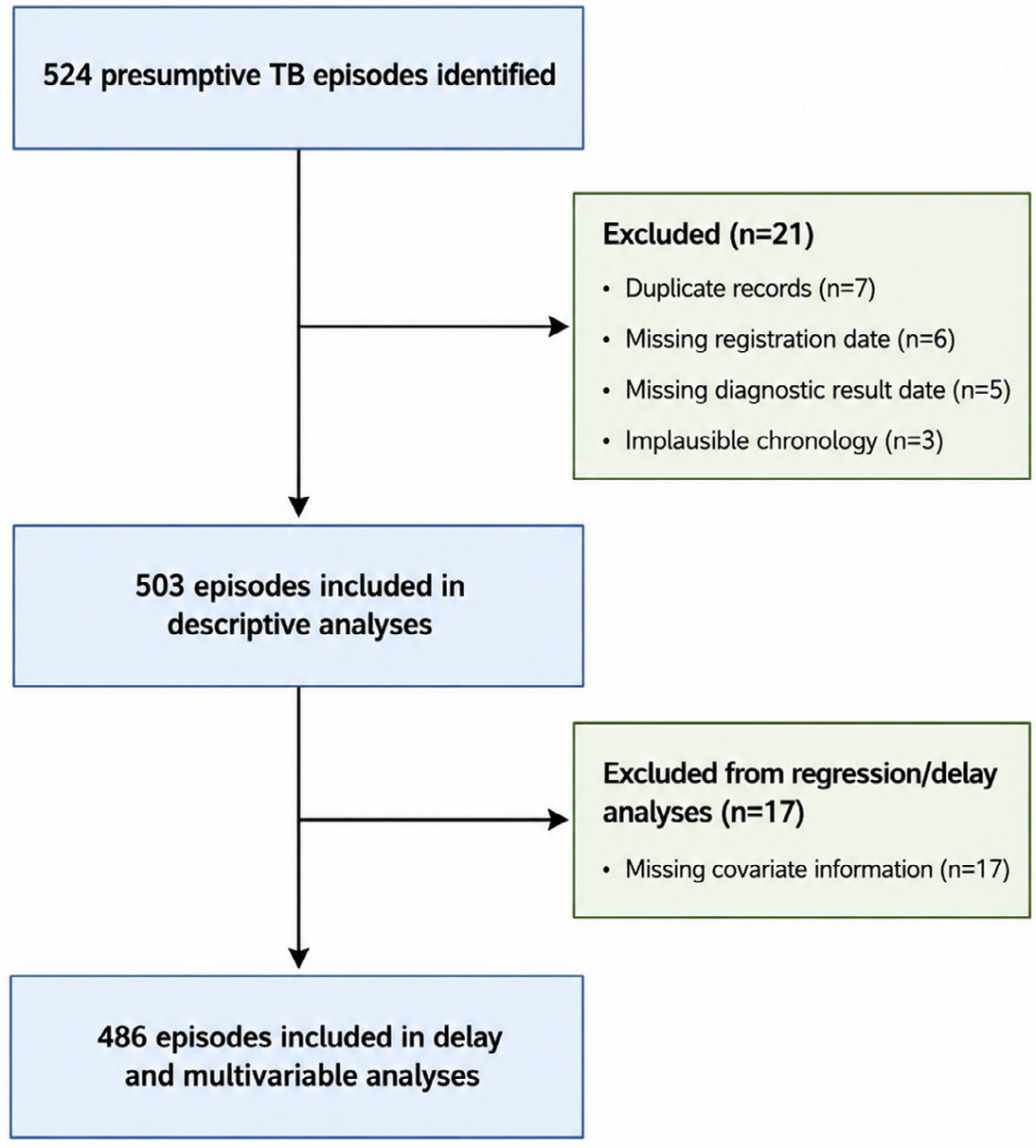
Selection of presumptive tuberculosis episodes.

Episodes were excluded if duplicate records, missing registration dates, missing diagnostic result dates, or implausible chronological sequences were identified during data cleaning. Additional records with missing covariate information were excluded from multivariable analyses. The selection process and reasons for exclusion are presented in Figure 1. The study population comprised all people entered into the presumptive tuberculosis register during the study period. A presumptive tuberculosis episode was defined as a person with clinical or epidemiological suspicion of tuberculosis who was assigned a presumptive tuberculosis registration number. For the present analysis, episodes were eligible if at least one diagnostic test had been ordered and documented according to the national tuberculosis diagnostic algorithm. Individuals registered as presumptive tuberculosis cases but without any documented diagnostic test order were not included because the primary outcome required a measurable interval between registration and the first recorded diagnostic result. Episodes with duplicate records, missing key dates, or implausible chronological sequences were excluded during data cleaning [4]. Episodes were excluded from delay analyses if registration or diagnostic dates were missing, duplicated, or implausible.

### Data cleaning and missing data

Data quality checks were performed before analysis. Duplicate records were identified using presumptive tuberculosis registration numbers and demographic information. Records with missing registration dates, missing diagnostic result dates, or implausible chronological sequences (for example, diagnostic results recorded before registration) were excluded from delay analyses. Missing covariate information was handled using complete-case analysis for multivariable models. The numbers of exclusions at each stage are presented in Figure 1.

### Variables and outcomes

Variables included age, sex, residential area, referral source, anatomical disease site, previous tuberculosis treatment status, documented diabetes, first diagnostic test, diagnostic result, final diagnosis, and treatment initiation. Diagnostic tests recorded in the register included sputum smear microscopy, Xpert MTB/RIF (GeneXpert), chest radiography, mycobacterial culture, histopathology, and other investigations performed according to the Indonesian National Tuberculosis Diagnostic Algorithm. The first diagnostic test variable referred to the earliest documented investigation requested following presumptive tuberculosis registration.

Age was analysed continuously and in life-course groups: 0–14, 15–24, 25–44, 45–64, and 65 years or older. The primary outcome was diagnostic delay, defined as the number of days from registration as a presumptive tuberculosis case to the first recorded diagnostic result. When more than one diagnostic result was recorded, the earliest valid result was used. Same-day results were assigned a delay of zero days. Prolonged delay was defined as more than 14 days, with more than 30 days assessed in sensitivity analyses. The >14-day threshold was selected because it has been commonly used in studies evaluating tuberculosis diagnostic delays and represents a pragmatic operational benchmark for identifying potentially avoidable delays within the diagnostic pathway. A >30-day threshold was examined in sensitivity analyses to assess more substantial delays and facilitate comparison with previous tuberculosis delay literature. Secondary outcomes were receipt of a final tuberculosis diagnosis among presumptive episodes and documented treatment initiation among diagnosed patients. This measure captures the interval after entry into the formal diagnostic system and may therefore be interpreted as a facility-level diagnostic turnaround indicator.

### Statistical analysis

Characteristics were summarised using medians and interquartile ranges or frequencies and percentages. Group differences were assessed using the Mann-Whitney U, Kruskal-Wallis, chi-square, or Fisher exact tests, as appropriate. Negative binomial regression estimated incidence rate ratios for diagnostic delay because the outcome represented count data (days) with a right-skewed distribution. Overdispersion was assessed by comparing the variance and mean of the delay variable and was observed to be present; therefore, negative binomial regression was preferred over Poisson regression. Logistic regression estimated adjusted odds ratios for prolonged delay, final diagnosis, and treatment initiation. Models included age group, sex, anatomical disease site, referral source, diabetes, and residence. Sensitivity analyses used the more than 30-day delay threshold and excluded records with missing key covariates. Statistical significance was assessed using two-sided tests with alpha = 0.05. Statistical analyses were conducted using Stata version 18.0 (StataCorp LLC, College Station, TX, USA).

### Ethics and consent

The study was approved by the Health Research Ethics Committee, Faculty of Public Health, Universitas Muhammadiyah Jakarta (approval number: 10.166.C/KEPK-FKMUMJ/I/2026; approval date: 30 January 2026). Permission to access and analyse register data was obtained from the Tasikmalaya City Health Office and the participating primary health centre. Direct identifiers were removed before analysis. Individual informed consent was waived because the study used retrospective, de-identified secondary data consistent with Indonesian health data governance requirements [17, 18].

## Results

The register contained 524 presumptive tuberculosis episodes. After excluding 21 records because of duplication, missing key dates, or implausible chronology, 503 episodes remained for descriptive analyses. A further 17 episodes with incomplete covariate information were excluded from multivariable analyses, resulting in a final analytic sample of 486 episodes for delay and regression analyses (Figure 1). Median age was 42 years (interquartile range, 27–58), 282 participants (56.1%) were male, 438 episodes (87.1%) involved pulmonary disease, and 226 episodes (44.9%) were self-presenting (Table 1). Age group and referral source differed by sex.

**Table 1:** Demographic and clinical characteristics of people with presumptive tuberculosis, 2025.

CHARACTERISTIC	TOTAL, *N* (%)	MALE, *N* (%)	FEMALE, *N* (%)	*P* VALUE
Total	503 (100.0)	282 (56.1)	221 (43.9)	
Age, median (IQR), years	42 (27–58)	45 (30–60)	39 (24–55)	0.018
0–14 years	48 (9.5)	25 (8.9)	23 (10.4)	
15–24 years	72 (14.3)	36 (12.8)	36 (16.3)	
25–44 years	147 (29.2)	78 (27.7)	69 (31.2)	
45–64 years	158 (31.4)	95 (33.7)	63 (28.5)	
>=65 years	78 (15.5)	48 (17.0)	30 (13.6)	0.041
Pulmonary disease	438 (87.1)	250 (88.7)	188 (85.1)	
Extrapulmonary disease	65 (12.9)	32 (11.3)	33 (14.9)	0.214
Self-presenting	226 (44.9)	137 (48.6)	89 (40.3)	
Community cadre referral	137 (27.2)	70 (24.8)	67 (30.3)	
Other health facility referral	96 (19.1)	52 (18.4)	44 (19.9)	
Private provider referral	44 (8.7)	23 (8.2)	21 (9.5)	0.032
Diabetes mellitus recorded	67 (13.3)	42 (14.9)	25 (11.3)	0.236
Inside catchment area	421 (83.7)	238 (84.4)	183 (82.8)	0.638

IQR = interquartile range. Percentages use column denominators unless otherwise indicated. *P* values compare male and female participants using Mann-Whitney U, chi-square, or Fisher exact tests.

Median diagnostic delay was 4 days (interquartile range, 1–11 days); 96 participants (19.8%) had same-day results. Delays longer than 14 days and 30 days occurred in 94 episodes (19.3%) and 37 episodes (7.6%), respectively. Delay varied by age group, anatomical disease site, and referral source (Table 2). The longest delays were observed among adults aged 65 years or older, people with extrapulmonary disease, and those referred from another health facility or private provider.

**Table 2:** Diagnostic delay by demographic and clinical characteristics.

CHARACTERISTIC	*N*	MEDIAN DAYS (IQR)	>14 DAYS, *N* (%)	>30 DAYS, *N* (%)	*P* VALUE
Total	486	4 (1–11)	94 (19.3)	37 (7.6)	
Male	273	5 (1–12)	58 (21.2)	24 (8.8)	
Female	213	3 (1–9)	36 (16.9)	13 (6.1)	0.086
0–14 years	46	2 (0–7)	5 (10.9)	2 (4.3)	
15–24 years	69	3 (1–8)	9 (13.0)	3 (4.3)	
25–44 years	143	4 (1–10)	24 (16.8)	8 (5.6)	
45–64 years	153	5 (2–13)	35 (22.9)	15 (9.8)	
>=65 years	75	7 (2–16)	21 (28.0)	9 (12.0)	0.011
Pulmonary disease	424	4 (1–10)	75 (17.7)	27 (6.4)	
Extrapulmonary disease	62	8 (3–18)	19 (30.6)	10 (16.1)	0.004
Self-presenting	219	3 (1–8)	31 (14.2)	10 (4.6)	
Community cadre referral	133	4 (1–10)	24 (18.0)	8 (6.0)	
Other health facility referral	92	7 (2–16)	25 (27.2)	12 (13.0)	
Private provider referral	42	8 (3–19)	14 (33.3)	7 (16.7)	0.002

IQR = interquartile range. Diagnostic delay was measured from presumptive tuberculosis registration to the first recorded diagnostic result. *P* values compare subgroup distributions of delay.

In adjusted negative binomial regression, age 65 years or older was associated with longer delay compared with age 25–44 years (adjusted incidence rate ratio, 1.46; 95% CI, 1.12–1.90; *P* = 0.005). Extrapulmonary disease (adjusted incidence rate ratio, 1.58; 95% CI, 1.21–2.07; *P* = 0.001) and referral from another health facility (adjusted incidence rate ratio, 1.35; 95% CI, 1.01–1.82; *P* = 0.045) were also associated with longer delay (Table 3). Male sex was not independently associated with delay.

**Table 3:** Negative binomial regression analysis of factors associated with diagnostic delay (n = 486).

VARIABLE	CRUDE IRR	95% CI	*P* VALUE	ADJUSTED IRR*	95% CI	*P* VALUE
**Sex**						
Male	1.18	0.98–1.42	0.081	1.12	0.93–1.36	0.229
Female	Reference	–	–	Reference	–	–
**Age group (years)**						
0–14	0.74	0.51–1.08	0.119	0.78	0.53–1.15	0.205
15–24	0.82	0.61–1.10	0.184	0.85	0.63–1.15	0.292
25–44	Reference	–	–	Reference	–	–
45–64	1.24	1.01–1.53	0.044	1.21	0.98–1.50	0.077
≥65	1.53	1.18–1.98	0.001	1.46	1.12–1.90	0.005
**Anatomical disease site**						
Extrapulmonary disease	1.71	1.33–2.20	<0.001	1.58	1.21–2.07	0.001
Pulmonary disease	Reference	–	–	Reference	–	–
**Referral source**						
Community cadre referral	1.18	0.91–1.52	0.210	1.13	0.87–1.47	0.360
Other health facility referral	1.49	1.12–1.98	0.006	1.35	1.01–1.82	0.045
Private provider referral	1.67	1.15–2.43	0.007	1.44	0.98–2.11	0.064
Self-presenting	Reference	–	–	Reference	–	–

Adjusted for age group, sex, anatomical disease site, referral source, diabetes mellitus status, and residential area. Negative binomial regression was used because diagnostic delay (days) was a count outcome with evidence of overdispersion. Diagnostic delay was defined as the interval between presumptive tuberculosis registration and the first recorded diagnostic result.

Logistic regression showed similar patterns for prolonged diagnostic delay (Table 4). Age 65 years or older (adjusted odds ratio, 1.88; 95% CI, 1.02–3.47; *P* = 0.043), extrapulmonary disease (adjusted odds ratio, 2.14; 95% CI, 1.15–3.98; *P* = 0.016), and referral from another health facility (adjusted odds ratio, 1.86; 95% CI, 1.03–3.35; *P* = 0.039) were independently associated with more than 14-day delay. The more than 30-day sensitivity analysis showed the same direction of association, although some estimates were less precise.

**Table 4:** Adjusted logistic regression analysis of prolonged diagnostic delay (n = 486).

VARIABLE	>14-DAY DELAY aOR*	95% CI	*P* VALUE	>30-DAY DELAY aOR*	95% CI	*P* VALUE
**Sex**						
Male	1.19	0.74–1.92	0.473	1.31	0.66–2.61	0.438
Female	Reference	–	–	Reference	–	–
**Age group (years)**						
0–14	0.64	0.23–1.79	0.394	0.77	0.16–3.70	0.742
15–24	0.73	0.31–1.71	0.468	0.75	0.19–2.91	0.674
25–44	Reference	–	–	Reference	–	–
45–64	1.42	0.80–2.52	0.229	1.58	0.67–3.71	0.296
≥65	1.88	1.02–3.47	0.043	2.03	0.82–5.03	0.126
**Anatomical disease site**						
Extrapulmonary disease	2.14	1.15–3.98	0.016	2.63	1.16–5.95	0.020
Pulmonary disease	Reference	–	–	Reference	–	–
**Referral source**						
Community cadre referral	1.22	0.69–2.16	0.494	1.18	0.46–3.00	0.730
Other health facility referral	1.86	1.03–3.35	0.039	2.21	0.94–5.22	0.069
Private provider referral	2.09	0.98–4.45	0.056	2.47	0.86–7.08	0.094
Self-presenting	Reference	–	–	Reference	–	–

Abbreviations: aOR, adjusted odds ratio; CI, confidence interval.

Multivariable logistic regression models were used to evaluate factors associated with prolonged diagnostic delay. The primary analysis defined prolonged delay as >14 days, while a >30-day threshold was examined in sensitivity analyses. Models were adjusted for age group, sex, anatomical disease site, referral source, diabetes mellitus status, and residential area. Reference categories were female sex, age 25–44 years, pulmonary disease, and self-presentation.

Overall, 176 presumptive episodes (35.0%) resulted in a tuberculosis diagnosis, including 118 bacteriologically confirmed drug-susceptible cases, 9 drug-resistant cases, and 49 clinically diagnosed cases. Treatment initiation was documented for 158 diagnosed patients (89.8%). Male sex was not significantly associated with final diagnosis or treatment initiation. Extrapulmonary disease was associated with lower odds of final diagnosis, and more than 14-day delay showed a non-significant tendency towards lower treatment initiation (Table 5).

**Table 5:** Distribution of tuberculosis diagnostic outcomes and treatment initiation.

OUTCOME	*N* (%)
**Among all presumptive tuberculosis episodes (*n* = 503)**	
Tuberculosis diagnosis	176 (35.0)
Not tuberculosis	295 (58.6)
Inconclusive or unknown	32 (6.4)
**Among diagnosed tuberculosis cases (*n* = 176)**	
Bacteriologically confirmed drug-susceptible tuberculosis	118 (67.0)
Bacteriologically confirmed drug-resistant tuberculosis	9 (5.1)
Clinically diagnosed tuberculosis	49 (27.8)
**Treatment initiation among diagnosed patients (*n* = 176)**	
Treatment initiation documented	158 (89.8)
No documented treatment initiation	18 (10.2)

Abbreviation: TB, tuberculosis.

In adjusted analyses, extrapulmonary disease was associated with lower odds of receiving a final tuberculosis diagnosis (adjusted OR, 0.58; 95% CI, 0.32–0.96; *P* = 0.035), whereas male sex was not significantly associated with tuberculosis diagnosis (adjusted OR, 1.39; 95% CI, 0.96–2.01; *P* = 0.081) (Table 6).

**Table 6:** Predictors of tuberculosis diagnosis.

VARIABLE	ADJUSTED OR	95% CI	*P* VALUE
Male sex	1.39	0.96–2.01	0.081
Extrapulmonary disease	0.58	0.32–0.96	0.035

Among patients diagnosed with tuberculosis, male sex was not significantly associated with treatment initiation (adjusted OR, 0.83; 95% CI, 0.30–2.27; *P* = 0.713). Diagnostic delay exceeding 14 days showed a non-significant tendency towards lower treatment initiation (adjusted OR, 0.46; 95% CI, 0.16–1.35; *P* = 0.158) (Table 7).

**Table 7:** Predictors of treatment initiation among patients diagnosed with tuberculosis (n = 176).

VARIABLE	ADJUSTED OR	95% CI	*P* VALUE
Male sex	0.83	0.30–2.27	0.713
Diagnostic delay >14 days	0.46	0.16–1.35	0.158

Abbreviations: OR, odds ratio; CI, confidence interval.

*Note:* Models were adjusted for age group, sex, anatomical disease site, referral source, diabetes mellitus status, and residential area, as appropriate. The treatment initiation model was restricted to participants with a confirmed tuberculosis diagnosis (n = 176).

## Discussion

### Principal findings

This register-based study found that diagnostic results were generally recorded within a short period after presumptive tuberculosis registration in primary care, but nearly one in five episodes still experienced a delay beyond 14 days. Older age, extrapulmonary disease, and referral from another health facility were the clearest predictors of longer post-registration delay. These findings suggest that even after a person enters the formal tuberculosis diagnostic pathway, specific patient groups and referral pathways remain vulnerable to avoidable diagnostic friction.

### Interpretation in relation to prior evidence

The median post-registration delay observed in this study was shorter than estimates from studies measuring time from symptom onset or first care seeking. Systematic reviews and Indonesian pathway studies have reported much longer total diagnostic intervals, often reflecting the cumulative effects of symptom appraisal, repeated provider visits, cost, stigma, and navigation across public and private services [7, 8, 19]. The present study, therefore, should not be interpreted as measuring total patient delay. Rather, it isolates a later operational segment: the time from formal presumptive tuberculosis registration in primary care to the availability of the first recorded diagnostic result.

### Equity implications for older adults and extrapulmonary tuberculosis

Older adults had longer delays, which is clinically plausible and programmatically important. Tuberculosis in older people may present with non-specific symptoms, coexisting chronic diseases, frailty, or difficulty producing sputum, all of which can reduce diagnostic suspicion or slow specimen collection and follow-up [20]. Primary health centre teams could therefore flag older presumptive patients for active tracking when results are not available within two weeks. Extrapulmonary disease was also associated with longer delay. The finding that extrapulmonary disease was associated with lower odds of receiving a final tuberculosis diagnosis warrants particular attention. Extrapulmonary tuberculosis frequently requires imaging, histopathology, or specialist assessment that may not be available within primary care facilities. Some patients may therefore be referred for further evaluation without complete documentation returning to the primary care register. This could contribute both to genuine diagnostic complexity and to under-ascertainment of confirmed diagnoses within routine primary care records. This aligns with evidence that extrapulmonary tuberculosis is more difficult to confirm because symptoms are site-specific, microbiological yield may be low, and diagnosis may require imaging, tissue sampling, or referral to higher-level facilities [21]. Clear escalation pathways for suspected extrapulmonary tuberculosis are needed so that referral does not become an unmonitored waiting period.

### Referral pathways and continuity of care

Referral source mattered. Patients entering from other health facilities had longer delays than self-presenting patients, and private-provider referrals showed elevated but less precise associations. Indonesian studies show that people with tuberculosis commonly seek care from private clinics, hospitals, pharmacies, or informal providers before reaching public tuberculosis services [7, 22]. These findings are highly relevant to public-private mix strategies because referral alone is not sufficient unless diagnostic requests, specimen movement, result return, and treatment linkage are actively closed [23, 24]. Standardised referral forms, date-stamped specimen requests, interoperable reporting, and feedback mechanisms may reduce losses between providers.

An additional continuity-of-care concern relates to treatment initiation after diagnosis. In this study, 18 of 176 diagnosed patients (10.2%) had no documented treatment initiation. Although the available data could not determine whether these individuals failed to start treatment or whether treatment initiation was not recorded, this finding may represent potential initial loss to follow-up. Patients who do not promptly initiate treatment remain at risk of disease progression, ongoing transmission, and disengagement from tuberculosis care services. Strengthening linkage-to-care mechanisms and treatment documentation systems may help reduce these losses within the tuberculosis care cascade.

### Global health relevance

From a global health perspective, the study highlights a practical equity-monitoring opportunity within routine primary care systems. Many high-burden countries rely on primary care registers and referral networks to close tuberculosis detection gaps. Register-based indicators can be used not only to count presumptive cases and confirmed diagnoses, but also to detect where specific groups experience slower diagnostic progression. This is especially relevant in lower- and middle-income countries, where diagnostic decentralisation, molecular testing access, and public-private coordination remain uneven [25–28]. Accordingly, our findings should not be interpreted as total tuberculosis diagnostic delay but rather as delay occurring after registration within the formal diagnostic pathway.

### Implications of diagnostic delays

Although the median diagnostic interval was relatively short, prolonged delays remain clinically and programmatically important. Delays in obtaining diagnostic results may postpone treatment initiation among patients ultimately diagnosed with tuberculosis, increase opportunities for initial loss to follow-up, and prolong periods during which infectious individuals remain untreated within households and communities. Delayed diagnostic results may also postpone the identification and management of alternative conditions among patients who do not have tuberculosis, potentially contributing to continued morbidity and repeated healthcare utilisation. In addition, prolonged waiting times may reduce patient confidence in health services and increase the likelihood of disengagement from care. These consequences highlight why monitoring diagnostic timeliness remains an important component of tuberculosis care cascade improvement, even after individuals have entered the formal diagnostic system.

### Strengths and limitations

The main strength of this study is its use of routine presumptive tuberculosis register data, which captures an operational point that is often missed in studies restricted to confirmed patients. An additional limitation relates to the use of routine register data. The primary outcome measured the interval from presumptive tuberculosis registration to the first recorded diagnostic result rather than the exact time at which a diagnostic result became available. Consequently, the observed interval may reflect delays in documentation, reporting, or data entry in addition to clinical or health-system diagnostic processes. The analysis also examined multiple care cascade outcomes, including diagnostic delay, final diagnosis, and treatment initiation. However, several limitations should be considered. First, the findings cannot be interpreted as comprehensive programme performance because the study was conducted in one primary health centre. Second, the register-based design measured delay from registration to diagnostic result and did not capture symptom onset, pre-registration care seeking, cost, stigma, distance, or provider switching. Third, routine registers may contain missing or inconsistently updated referral, comorbidity, and treatment fields. In addition, HIV status was incompletely recorded in the routine register and could not be reliably incorporated into multivariable analyses. Consequently, potential confounding related to HIV infection could not be fully assessed. Finally, because the study was conducted in a single primary health centre, the findings should be interpreted primarily as locally relevant operational evidence rather than as representative of Indonesian primary care tuberculosis services nationally. In addition, the register did not consistently capture the specific diagnostic modality associated with each recorded result. Consequently, it was not possible to determine which diagnostic tests contributed to same-day (0-day) results or to evaluate the extent to which variation in diagnostic turnaround time reflected differences in testing technologies. Because diagnostic modalities such as Xpert MTB/RIF, smear microscopy, culture, and other investigations have substantially different turnaround times, unmeasured variation related to diagnostic test type may have influenced the observed delay estimates.

## Conclusions

Diagnostic results were usually recorded rapidly after presumptive tuberculosis registration, but delays remained concentrated among older adults, people with extrapulmonary disease, and referred patients. Because prolonged diagnostic delays may contribute to treatment postponement, initial loss to follow-up, and continued transmission, strengthening register-based tracking, referral feedback mechanisms, and escalation pathways for suspected extrapulmonary tuberculosis may support more equitable continuity across the primary care tuberculosis care cascade.

## Data Availability

The individual-level register data analysed in this study are not publicly available because they originate from routine health service records and are subject to local data governance and privacy restrictions. De-identified aggregate outputs may be available from the corresponding author upon reasonable request and with approval from the relevant health authority and ethics committee.
